# Supplementary material for: Cyclosporines Antagonize the Antiviral Activity of IFITMProteins by Redistributing Them toward the Golgi Apparatus
Source: Biomolecules. 2023 Jun 3;13(6):937. doi: 10.3390/biom13060937 (PMC10296495; doi:10.3390/biom13060937)
Supplement: Supplementary file 1 [file biomolecules-13-00937-s001.zip › biomolecules-2430342-supplementary.pdf]

Supplementary Materials

Plate #	Background	STD	IAVpp	STD	S/B	Z'
Plate 1	1.09	0.02	4.93	0.14	4.54	0.88
Plate 2	1.04	0.04	4.81	0.10	4.65	0.89
Plate 3	1.19	0.05	4.69	0.10	3.95	0.87
Plate 4	1.19	0.05	4.78	0.11	4.03	0.87

Table S1. Validation of the BlaM virus-cell fusion assay for high-throughput screening of LOPAC library. MDCK.Vector cells showed a robust fusion signal with S/B ratio of ~5 and Z' of 0.8 for all four 384-well plates, indicating a high-quality assay for the screen

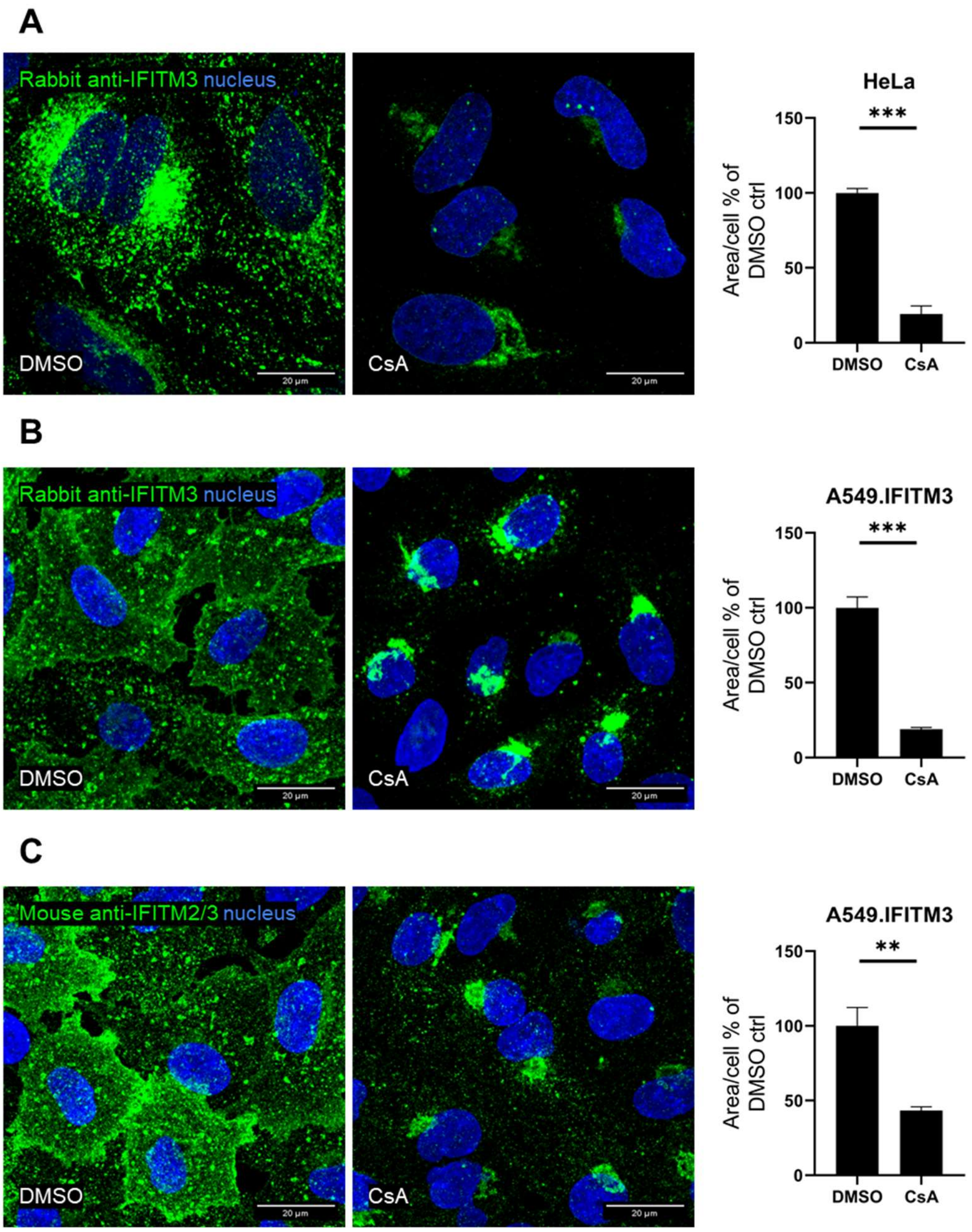

Figure S1. Immunofluorescence analysis of CsA-induced IFITM3 relocalization using different anti-IFITM3 antibodies. HeLa cells (A) or A549.IFITM3 cells (B, C) were pretreated with CsA (20 μM) or a vehicle control (DMSO) for 1.5 hr, fixed, and stained using rabbit produced anti-IFITM3 (A, B) or mouse produced anti-IFITM2/3 (C) for IFITM3 and GM130. For each sample, 3 image fields were acquired. The area covered by IFITM's signal was determined as in Fig. 2. Statistical analysis was done using Student's *t*-test. ***, $p < 0.001$.

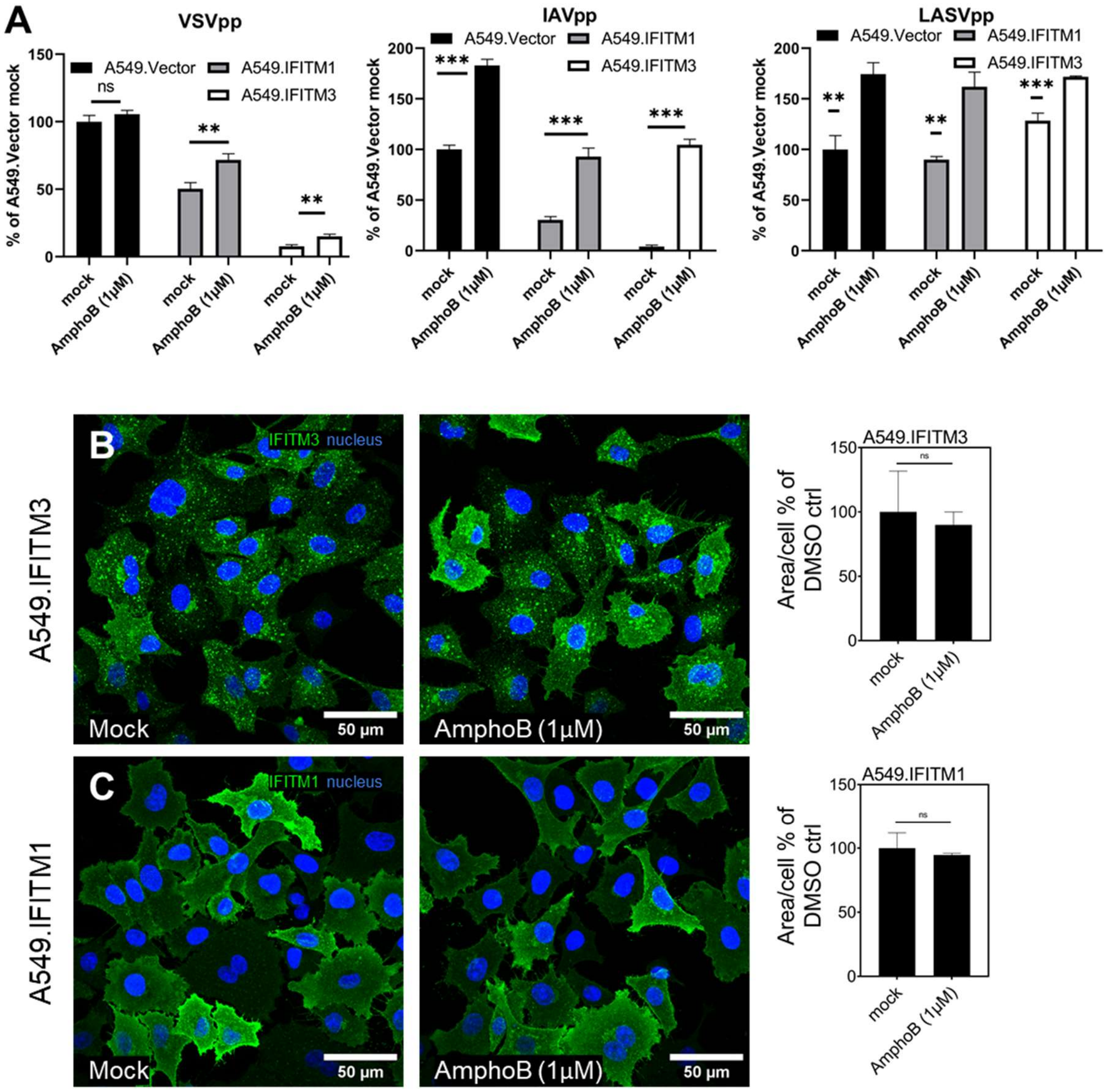

Figure S2. Amphotericin B rescues viral fusion in IFITM-expressing cells without inducing its relocalization. A549 cells transduced with an empty vector, IFITM1 or IFITM3 expressing vector were pretreated with Amphotericin B (1 μM) or mock-treated medium for 1.5 hr followed by infection with BlaM-Vpr containing pseudoparticles (A). A549.IFITM3 (B) and A549.IFITM1 (C) cells were treated as in (A), fixed, and stained for IFITM3 or IFITM1 together with Golgi marker. For each sample, 3 image fields were acquired. The JaCoP ImageJ plugin was used to measure respective IFITM and GM130 colocalization. Statistical analysis was done using Student's *t*-test. *, $p < 0.05$; **, $p < 0.01$; ns, not significant.

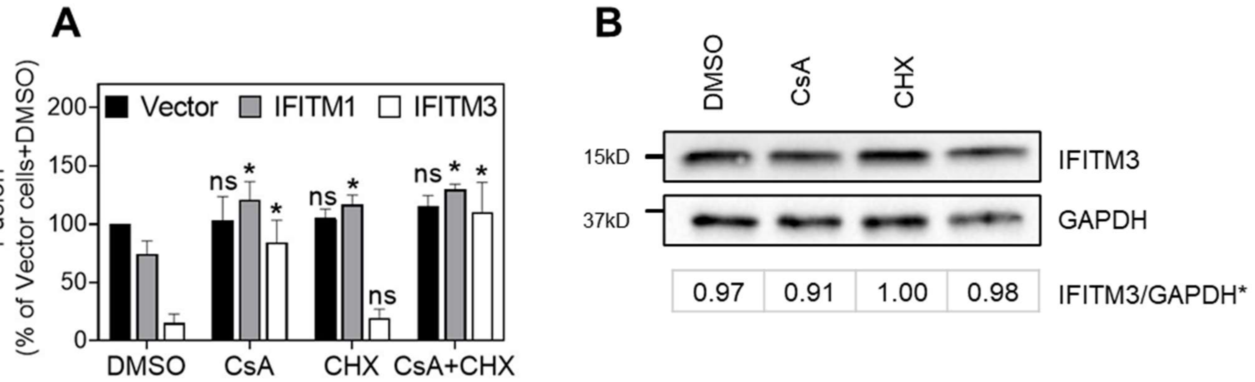

Figure S3. ***De novo*** **protein synthesis is not required for viral fusion rescue by CsA.** A549 cells transduced with an empty vector, IFITM1 or IFITM3 expressing vector were pretreated with DMSO, CsA (20 μM), CHX (10 μg/ml) or CsA and CHX 1.5 hr followed by inoculation with BlaM-Vpr containing IAVpp (A). A549.IFITM3 (B) were treated in the same manner as in (A), lysed, and whole cell lysate was analyzed by Western blotting. Statistical analysis was done using Student's *t*-test by comparing each sample to DMSO control *, $p < 0.05$; **; ns, not significant.

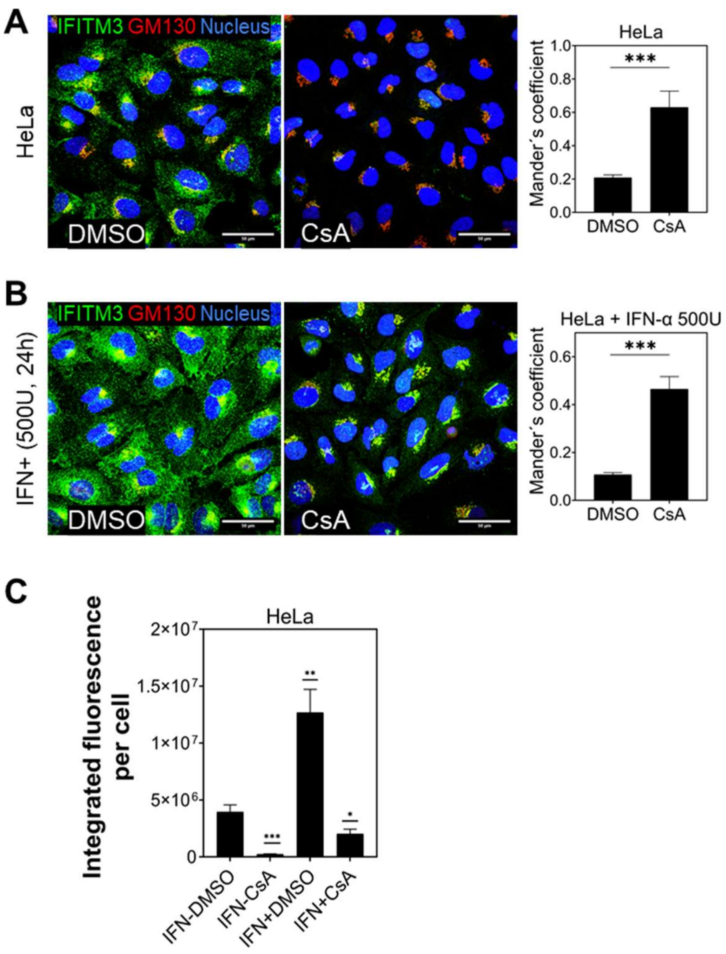

Figure S4. Cyclosporine A induces IFITM3 degradation in HeLa cells untreated with IFN alpha. HeLa cells pretreated (IFN+) or not (IFN-) with IFN were incubated with 20 µM CsA for 90 min or left untreated. Cells were fixed, permeabilized and immunostained for IFITM3 and GM130. (A) Representative images of cells not exposed to IFN and analyses of IFITM3/Golgi colocalization before and after CsA treatment. (B) Representative images of cells pretreated with IFN and analysis of IFITM3/Golgi colocalization before and after CsA treatment (reproduced from Fig. 6I). For each sample, 3 image fields containing were acquired. The JaCoP ImageJ plugin was used to measure respective IFITM and GM130 colocalization. (C) Quantification of the effect of IFN treatment on the IFITM3 expression levels in HeLa cells was done using FIJI software to measure integrated fluorescence after background subtraction and normalized to the cell number per image field. Statistical analysis was done using Student's *t*-test. *, $p < 0.05$; **, $p < 0.01$; ***, $p < 0.001$; ns, not significant. See also Figure 6.

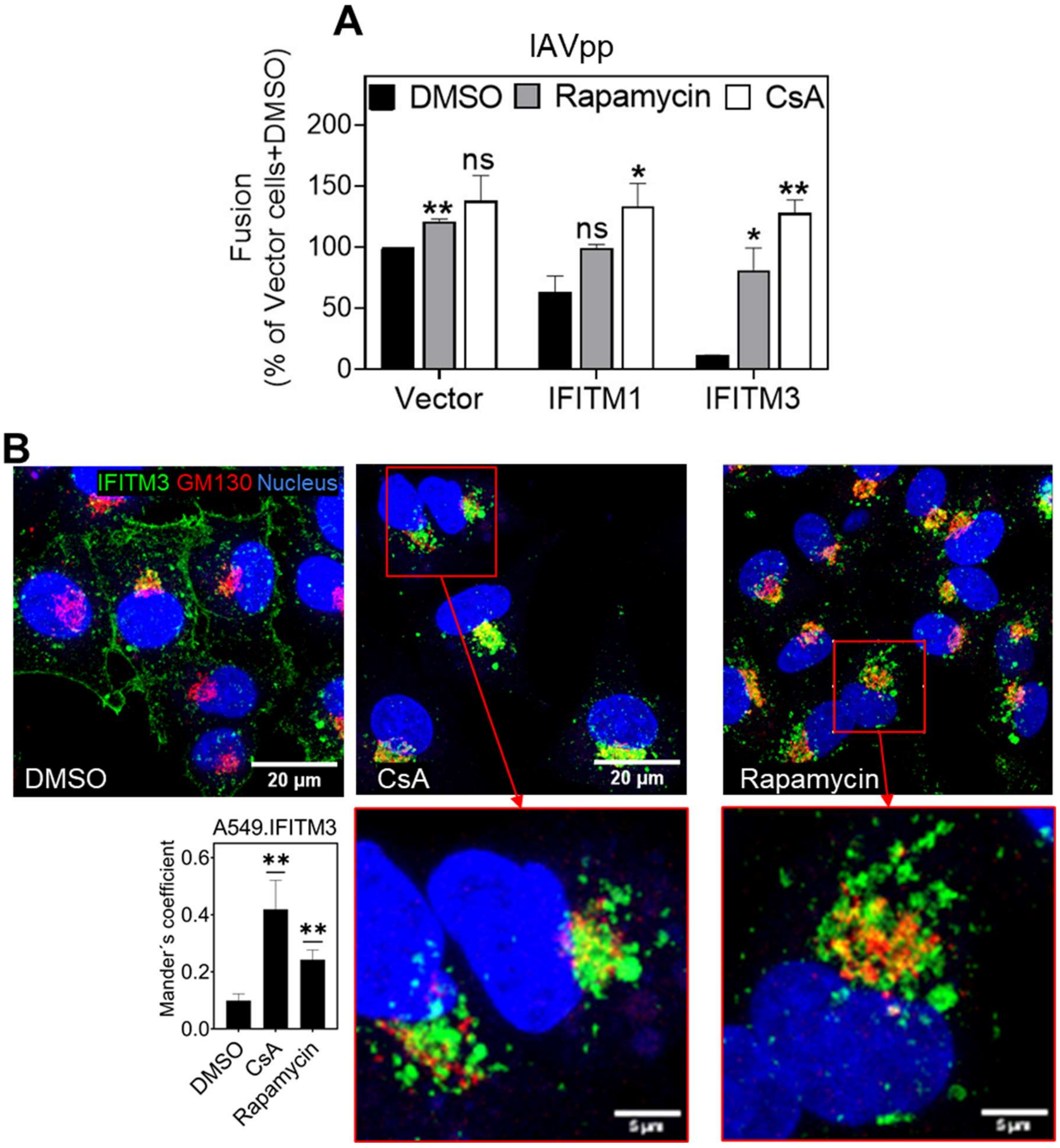

Figure S5. Rapamycin rescues IAVpp fusion with IFITM-expressing cells and promotes IFITM redistribution toward the Golgi area. A549.IFITM3 cells were incubated with DMSO, CsA, or Rapamycin (both at 20 μM) for 1.5 hr, fixed, and stained for IFITM3 and Golgi marker. For each sample, 3 image fields were acquired. The JaCoP ImageJ plugin was used to measure respective IFITM and GM130 colocalization. Statistical analysis was done using Student's *t*-test. *, $p < 0.05$; **, $p < 0.01$; ns, not significant.

Fig. S6

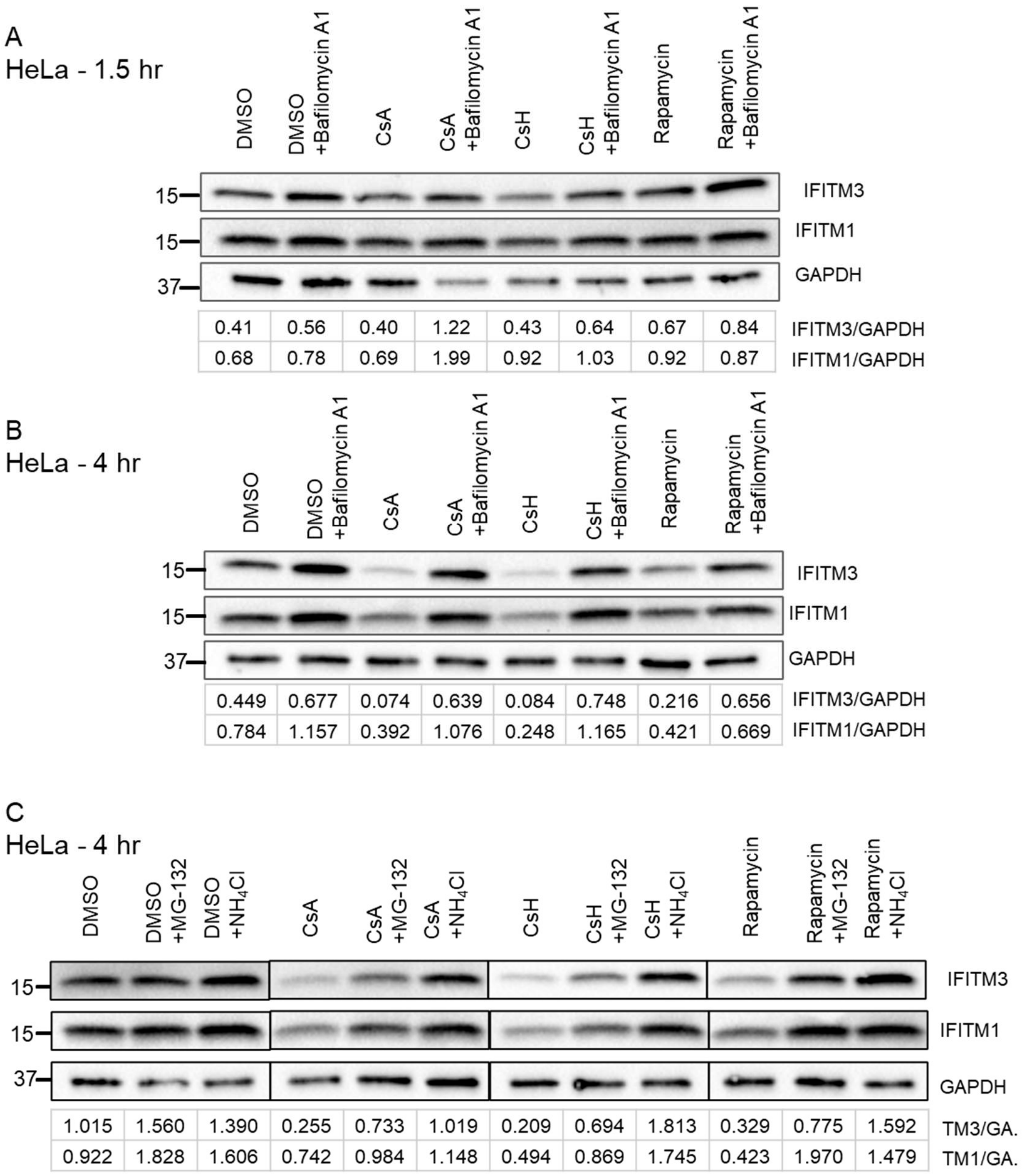

Figure S6. Analysis of IFITM degradation in the presence of cyclosporines and rapamycin. HeLa cells treated for 1.5 (A) or 4 hr (B) with DMSO, CsA, CsH, or Rapamycin (all at 20 μM) with or without Bafilomycin A1 (1μM), were lysed and whole cell lysate was analyzed. (C) HeLa cells were treated for 4 hr with DMSO, CsA, CsH, or Rapamycin (all at 20 μM) with or without MG-132 (10 μM) or NH_4Cl (40 mM) followed by cell lysis and analysis by SDS-PAGE and Western blotting.
